# Molybdenum-independent [4Fe-4S]-Catalyzed Sulfate Assimilation Sustains *Salmonella* Virulence

**DOI:** 10.21203/rs.3.rs-7730877/v1

**Published:** 2025-10-28

**Authors:** Ju-Sim Kim, Siva Uppalapati, Alyssa Margolis, Michael McClelland, Hanan Elajaili, Eva Nozik, Lin Liu, Andres Vazquez-Torres

**Affiliations:** 1 University of Colorado School of Medicine, Department of Immunology and Microbiology, Aurora, Colorado-80045, USA; 2 University of California Irvine School of Medicine, Department of Microbiology and Molecular Genetics, Irvine, CA, USA; 3 Cardiovascular Pulmonary Research Laboratories and Pediatric Critical Care Medicine, University of Colorado Anschutz Medical Campus, Aurora, Colorado-80045, USA; 4 Veterans Affairs, Eastern Colorado Health Care System, Aurora, Colorado-80045, USA

## Abstract

Nontyphoidal *Salmonella* exploits the metabolic versatility of its 17 molybdenum cofactor (MOCO)-containing MopB family members to respire on substrates as chemically disparate as formate, nitrate, and methionine sulfoxide^1–4^. Here, we identify three hitherto unknown periplasmic sulfate reductases within the *Salmonella* MopB family. These enzymes have departed from the conventional energetics of canonical MopB molybdoenzymes to embrace roles in the biosynthetic assimilation of sulfur. The three periplasmic sulfate reductases facilitate the formation of hydrogen sulfide and [Fe-S] metalloproteins, thereby enabling *Salmonella* to grow in gut and viscera while defending against reactive species engendered by the phagocyte NADPH oxidase. In unprecedented fashion, the catalytic cycle of these periplasmic sulfate reductases is independent of the MOCO metal center used by archetypical MopB enzymes such as nitrate or dimethyl sulfoxide reductases, relying instead on the redox activity of the nearby [4Fe-4S] prosthetic group. The existence of orthologs of *Salmonella* periplasmic sulfate reductases in distant evolutionary branches suggests that [4Fe-4S]-dependent catalysis may occur across the ubiquitous MopB superfamily. Our research in *Salmonella* offers insights into the modular evolution of redox centers in the widespread MopB superfamily, which has significantly shaped the biogeochemistry of Earth for over 2.5 billion years.

Bacterial pathogens balance metabolism and energetic outputs according to the terminal electron acceptors available in the host. Molecular oxygen (O_2_) is the most energetically favorable, and thus the preferred, terminal electron acceptor for facultative anaerobic bacteria such as *Salmonella enterica*^5^. However, *Salmonella* experiences O_2_ shortages in the host. Aerobic respiration by enterocytes lining the intestine dramatically curtails the concentration of O_2_ diffusing into the gut lumen^6^. Consumption of O_2_ in the respiratory burst of the phagocyte NADPH oxidase also induces hypoxia in the *Salmonella*-containing vacuole of macrophages^7,8^. With restricted O_2_ availability, nontyphoidal *Salmonella* must exploit thermodynamically less favorable terminal electron acceptors such as nitrate, tetrathionate or methionine sulfoxide^1,4^. Tetrathionate, nitrate and methionine sulfoxide reductases belong to the ancient DMSO reductase superfamily whose founding members were already present in the last universal common ancestor^9^. The DMSO reductase superfamily underwent significant diversification 2.5 billion years ago, providing the evolving prokaryotes and archaea with a panoply of energetic solutions to the redox and terminal electron acceptor revolution that arose following the introduction of O_2_ in the Great Oxidation Event (GOE) of the Paleoproterozoic Era. The *Salmonella* genome encodes four *dmsA* paralogs, whose function is currently unknown. In the following investigations, we undertook genetic, biochemical and biophysical approaches to identify the roles and substrate preference of these *dmsA* paralogs in nontyphoidal *Salmonella*.

## DmsA paralogs enable *Salmonella* to colonize the gut and viscera.

**Figure 1.**
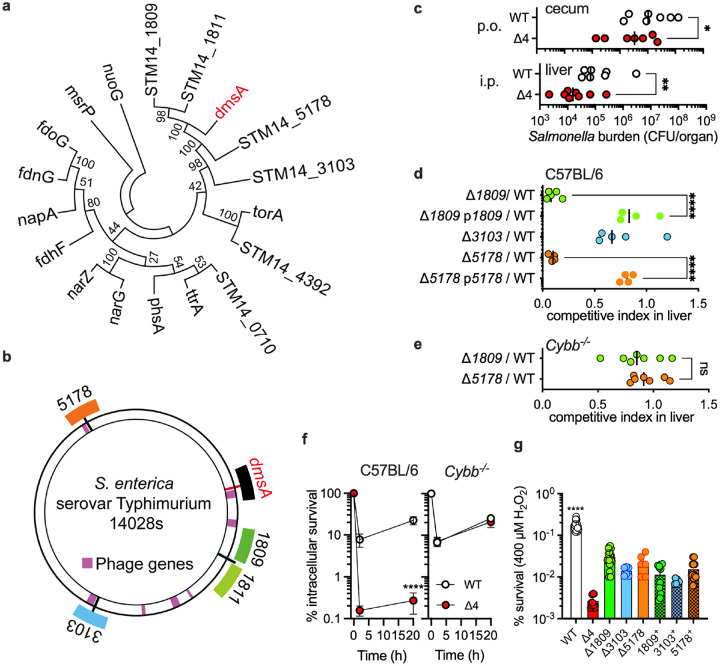
DmsA paralogs protect *Salmonella* against the phagocyte NADPH oxidase. **a.** Phylogenetic tree constructed using Neighbor-Joining in MEGAx software with 500 bootstrap replicates based on amino acid sequences of the DMSO reductase family members in the genome of *S. enterica* serovar Typhimurium strain 14028s. Branch lengths in the tree are proportional to evolutionary distances calculated using the Poisson correction method. Numbers in the tree represent the bootstrap values. **b**. Mapping of *dmsA* paralogs in the genome along with adjacent prophage elements. **c,** Δ4 indicates deletion of all four dmsA paralogs. **c, d, e** *Salmonella* strains were evaluated for virulence in C57BL/6 (**c, d**) and *Cybb*^*−/−*^ (**e**) mice. Bacterial fitness was assessed after 4 (**c**; p.o.) or 3 days (i.p., **d**, **e**) post-infection. Data are the mean ± SD (**c,** n=7,7,9,9; **d,** n=5,4,5,5,4; **e,** n=7,7). **f.** Percent survival of *Salmonella* in periodate-elicited macrophages from *C57BL/6* or *Cybb*^*−/−*^ mice with peritoneal exudate cells grown at 1% O_2_. The data are mean ± SD (C57BL/6 n=7; *Cybb*^−/−^ n=10). **g.** Survival of *Salmonella* after 2 h of treatment with 400 μM H_2_O_2_. Some strains expressed single dmsA paralogs (e.g., 1809^+^). Data are the mean ± SD (n=18,12,18,6,6,12,6,12). *, *p* < 0.5; **, *p* < 0.01; ****, *p* < 0.0001 by paired *t* test (**c**), unpaired *t* test (**d, e**), two-way ANOVA (**f**), or one-way ANOVA (**g** df =82,6, F = 18.79).

The *dmsA* gene (*STM14_1089)* encodes a novel methionine sulfoxide reductase within the DMSO reductase superfamily, while retaining its ability to metabolize DMSO^4^. The *Salmonella* genome encodes four additional *dmsA* paralogs organized in three distinct gene clusters, indicating a series of duplication events (Fig 1a, 1b, **S1a, Table S1**). The quintessential anaerobic Fumarate Nitrate Reduction (FNR) regulator is key to the adaptation of intracellular *Salmonella* to oxidative products of the phagocyte NADPH oxidase^10^, but the mechanisms by which this regulator shields *Salmonella* from the noxious effects of oxidative stress are incompletely understood. The four paralogs of *dmsA* (*STM14_1809*-, *STM14_1811*-, *STM14_3103*-, and *STM14_5178*) are among the most activated loci by FNR^10^. We hypothesized that *Salmonella* may utilize the uncharacterized *dmsA* paralogs during its associations with vertebrate hosts. To test this idea, we constructed a strain deficient in all four paralogs (designated “Δ*4”*). The Δ*4 Salmonella* strain was attenuated in C57BL/6 mice when inoculated orally (p.o.) or intraperitoneally (i.p.) together with equal numbers of wild-type isogenic bacteria (Fig 1c, **S1b, S1c**). Construction of defined mutants revealed that proteins encoded by *STM14_1809* and *STM14_5178* contributed to a greater extent to the fitness of *Salmonella* in systemic tissue than *STM14_1811* and *STM14_3103* (Fig 1d, **S2a, S2b**). The Δ*STM14_1809* and Δ*STM14_5178 Salmonella* strains recovered fitness in C57BL/6 mice when complemented with the low-copy pWSK29 plasmid bearing the *STM14_1811–1807* or *STM14_3103–3101* operons (Fig 1d, **S2c**). The *ΔSTM14_1809* and *ΔSTM14_5178* mutant strains also became virulent in *Cybb*^*−/−*^ mice (Fig 1e, **S2d**) and macrophages (Fig 1f, **S3a**) deficient in the phagocyte NADPH oxidase^11,12^. Consistent with this, *Salmonella* used the added functions of the four uncharacterized *dmsA* paralogs to resist bactericidal and bacteriostatic concentrations of hydrogen peroxide (H_2_O_2_) (Fig 1g, **S3b, S3c**). Together, these results demonstrate that the presence of additional *dmsA* family members has enhanced the capacity of *Salmonella* to withstand the oxidative stress of the phagocyte NADPH oxidase.

## DmsA paralogs encode novel periplasmic sulfate reductases.

**Figure 2.**
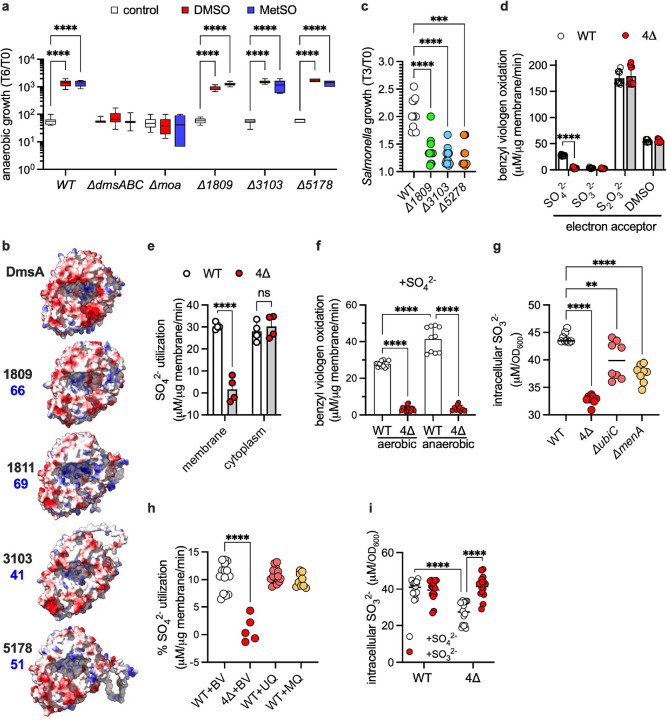
DmsA paralogs have novel periplasmic sulfate reductase activity. **a.** Anaerobic growth of *Salmonella* in MOPS-CAA medium containing 40 mM DMSO or L-methionine sulfoxide (L-MetSO). The data are the mean ± SD (n=12 *Δ5178*, and n=6 *Δ1809*). **b.** Surface electrostatic potential maps of DmsA paralogs were predicted using AlphaFold_3_ and visualized with UCSF ChimeraX. DmsA was used as the reference structure. Electrostatic surfaces are depicted with negative potentials in red and positive potentials in blue. The percentage identity of each paralogue relative to DmsA is shown in blue. **c.** Growth of *Salmonella* in MOPS-GLC medium at 37°C for 3 h under aerobiosis without shaking. Data are the mean ± SD; n = 12. df =11, F=25.61 **d, f.** Reductase activities in membranes isolated from *Salmonella* strains were measured spectrophotometrically at 570 nm by monitoring benzyl viologen oxidation in the indicated electron acceptor: DMSO (**d**), sulfate (**d, f**), sulfite (**d**), or thiosulfate (**d**). Membranes were obtained from bacteria grown in LB broth under aerobic (**f**) or anaerobic (**d,f**) conditions. The data are represented as the mean ± SD from 5–6 independent experiments (**d**, n=10; sulfate n=12; **f**, aerobic n=14, anaerobic n=10). **e.** Evaluation of sulfate reduction from the membrane and cytoplasm. Sulfate utilization was measured in isolated membrane and cytoplasmic fractions of *Salmonella*. Data represent mean ± SD; N = 4 from 2 individual experiments. **g, i.** Intracellular sulfite concentrations were quantified in *Salmonella* grown overnight in either MOPS-GLC minimal medium supplemented with sulfate (**g, i**) or sulfite (**i**) under ambient air without shaking. The data are the mean ± SD from 2 individual experiments (**g,** n=16; **i,** n=8). **h**. Sulfate reductase activity in isolated membranes was evaluated by quantifying sulfate utilization in the presence of different electron carriers. The enzyme activity demonstrated dependency on both ubiquinone (UQ) and menaquinone (MQ), while benzyl viologen (BV) served as a control. The data are the mean ± SD; n = 6 from 2–3 independent experiments. **, *p* < 0.01; ****, *p* < 0.0001 by two-way ANOVA (**a,d,e,f,g**) or by one-way ANOVA (**c, h, i**).

Based on the substrate specificity of DmsABC^4^, we tested whether the DmsA paralogs reduce methionine sulfoxide or DMSO. Strains bearing deletions in *STM14_1809, STM14_3103*, and *STM14_5178* gene clusters respired anaerobically on DMSO or methionine sulfoxide as efficiently as wild-type controls (Fig 2a), suggesting that the duplicated *dmsA* paralogs have assumed different function. Diversification of function is independently suggested by the fact that the DmsA paralogs share only about 40–70% amino acid identity (Fig 2b, **Table S1**), with notable charge differences in the funnel leading to the MOCO-catalytic site (Fig 2b). *STM14_1809, STM14_3103*, and *STM14_5178* mutant strains consistently grew poorly in MOPS-glucose (GLC) media when compared to wild-type controls (Fig 2c). Because sulfate is the sole sulfur compound in the MOPS-GLC media, we tested whether DmsA paralogs may facilitate anaerobic respiration on this naturally occurring and abundant oxyanion. To test this idea, we monitored the oxidation of the electron donor benzyl viologen by membranes isolated from wild-type or *dmsA* paralog-deficient *Salmonella*. Membranes isolated from the Δ4 *Salmonella* strain failed to respire on sulfate (*p* < 0.0001) (Fig 2d), whereas membranes expressing a single *dmsA* paralog supported intermediate levels of sulfate reductase activity (**Fig S4a**). Membranes from Δ*4 Salmonella* respired normally on thiosulfate or dimethyl sulfoxide (DMSO) (Fig 2d), demonstrating that other MOCO-dependent anaerobic respiration systems are fully functional. Accordingly, thiosulfate or hydrogen sulfide (H_2_S) supported the growth of the Δ*4 Salmonella* strain (**Fig S4b**). Cumulatively, these data indicate that *STM14_1809, STM14_3103*, and *STM14_5178* gene products encode hitherto unknown periplasmic sulfate reductases. Considering their phylogenetic relationships (Fig 1a, 1b) and their cellular location, these orphan operons will be renamed henceforth as *psr* for periplasmic sulfate reductases*: psr1A* for the gene *STM14_3103*, *psr2A* for *STM14_5178* and *psr3A* for *STM14_1809*.

Cytoplasmic extracts isolated from the Δ4 *Salmonella* strain reduced sulfate at wild-type levels (Fig 2e), indicating that *Salmonella* harbors sulfate reductase systems in both the membrane and cytoplasm. The sulfate reductase present in the membranes of aerobically grown *Salmonella* was induced further under anaerobiosis (Fig 2f), possibly reflecting transcription (**Fig S4c**). These findings indicate that membrane-bound periplasmic sulfate reductases are active aerobically and, more prominently, anaerobically. The expression of *psr* genes under aerobiosis and anaerobiosis raises the possibility that these novel sulfate reductases may use the reducing power of both ubiquinone and menaquinone. Accordingly, membranes from Δ*ubiC* or Δ*menA Salmonella*, which are deficient in key steps in the biosynthesis of ubiquinone or menaquinone, respectively^13^, harbored intermediate sulfate reductase levels (Fig 2g). The addition of ubiquinone or menaquinone supported excellent sulfate utilization by periplasmic sulfate reductases in a biochemically reconstituted *in vitro* system (Fig 2h). The Δ*4 Salmonella* strain growing in sulfate as the sole sulfur source contained lower levels of intracellular sulfite than wild-type controls (Fig 2i), indicating that the sulfate reductase activity present in the membrane of *Salmonella* initiates the two-electron reduction of sulfate to sulfite. The enzymology of the sulfate reductase present in the membrane appears, however, to be distinct from that of the cytoplasmic CysIJ pathway, in which reduction of sulfate to sulfite involves ATP-dependent synthesis of adenosine 5’phosphosulfate and 3’-phosphoadenosine 5’-phosphosulfate intermediates (**Fig S4d** and ref^14^). Accordingly, the addition of ATP did not improve the sulfate reductase activity present in *Salmonella* membranes (**Fig S4e**).

## Periplasmic sulfate reductases rely on the FS0 metal rather than the canonical MOCO prosthetic group.

**Figure 3.**
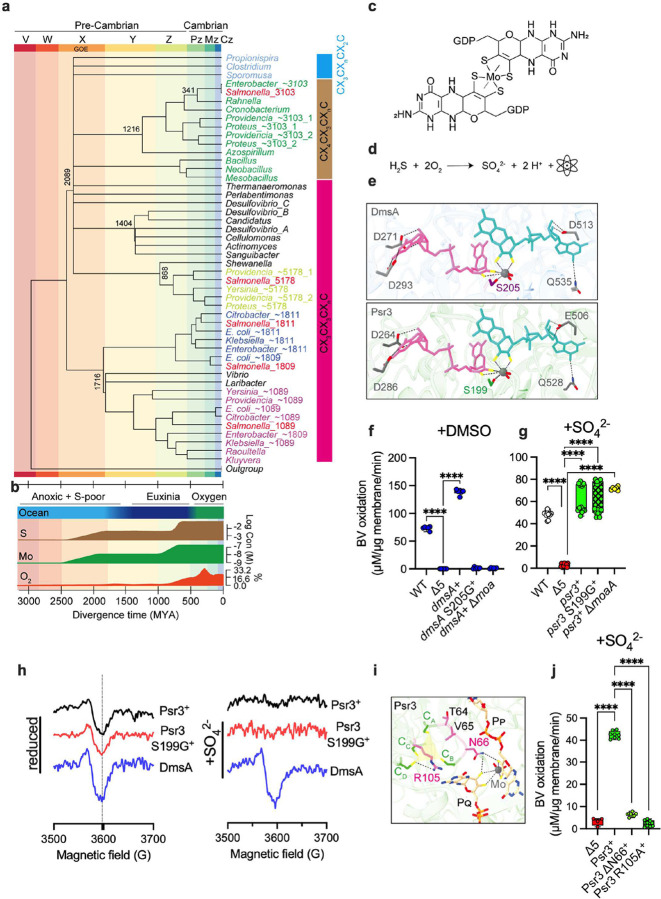
A novel iron-sulfur–dependent periplasmic sulfate reductase in *Salmonella* functions independently of molybdenum cofactor. **a.** Molecular timetree of DmsA-like proteins, overlaid with historical redox and Earth metal trends. Divergence times were inferred using RelTime and calibrated with known evolutionary events. **b.** Geochemical overlays showing atmospheric/oceanic S, Mo, and O_2_ levels over time. **c.** Structural representation of the Mo-bis PGD cofactor in the DmsA protein. **d.** Aerobic oxidation of H_2_S to sulfate is shown. The symbol presents energy. **e.** The predicted Mo-bis PGD is conserved in DmsA and Psr3. Coordinating serine residues (grey) and pterin (magenta and cyan)-interacting residues are shown. Distance to the active site is very similar (highlighted) in DmsA and the Psr3 paralog. **f, g, j.** The membranes used for EPR spectroscopy were evaluated DMSO and sulfate reductase activities by measuring the oxidation of benzyl viologen in the presence of DMSO (**f**) or sulfate (**g,i**). Δ5 is the quintuple deletion of *dmsA* and its four paralogs. The data are the mean ± SD from 2–7 independent experiments (**g,** n=6; **h,** WT n=12, Δ5=20, *psr3*^+^ n=14, *psr3 S199G*^+^ n=14, *psr3*^+^ Δ*moa* n=6; **i**, Δ5=4, n=8). ****, *p* < 0.0001 by one-way ANOVA (**f, g, j**). **h,** EPR spectra of FS0 [4Fe-4S] clusters were obtained from membranes reduced with 2 mM dithionite, either alone (**reduced**) or in combination with 50 mM sulfate (**SO4**^**2−**^). **i.** Structural model of Psr3 showing FS0 coordination relative to the Mo-bis PGD prosthetic group. Conserved carbon atoms and residues, Asn^66^ and Arg^105^, interact with the pterin ring system.

Molecular clock analysis placed the emergence of DmsA within archaeal lineages (Fig. 3a). The time tree topology reveals a previously underappreciated lineage composed of organisms from low-oxygen, sediment/host-linked niches, which include taxa bearing Psr2-type paralogs. The members of the *psr* homology group appear in many other genera, including distant *Proteus* (Fig. 3a), indicating that this operon is under strong selective pressure for its retention in some species. The lack of synteny in deeper branches—same *psr* but in different locations in the genome (**Fig. S5**)—likely indicates that lateral transfer occurred numerous times over 100 million years. Structural inspection of the [4Fe-4S] “FS0” coordination in the catalytic A subunit further partitions the tree into two dominant lineages. The group containing DmsA/Psr2A/Psr3A shares a characteristic **C3XC**3XCX_n_C FS0 architecture, whereas the ancestral Psr1A clade originating earlier in evolution bears a **C4XC**3XCX_n_C. The overall time-calibrated DmsA phylogeny resolves at least four major lineages and reveals a pronounced radiation in the late Archean (X) to early Paleoproterozoic (Y) Eras. This radiation event correlates with geological tectonic shifts and the percolation of sulfur and molybdenum into the ocean waters of the Neo-Archaean Earth (Fig 3b), two elements that are quintessential in the catalytic MOCO center (Fig 3c). Although significant diversity is already observed early in the evolution of DmsA homologs, a massive adaptive radiation event occurred in the Paleoproterozoic Era with the introduction of O_2_ on Earth (Fig 3a, 3b). Our phylogenetic analysis supports the idea that the photosynthetic synthesis of O_2_ on Earth, and the accumulation of novel electron acceptors such as thiosulfate, tetrathionate and sulfate (Fig 3d, ref^15^), were significant forces driving the massive diversification of the DMSO superfamily and the evolution of sulfate reductases^9^.

We used these phylogenetic analyses to gain insights into the atomic features underlying the sulfate reductase activity among members of the DMSO reductase superfamily. The catalytic molybdenum in DMSO reductases is hexacoordinated by the dithiolenes of the pterin guanosine dinucleotide, the hydroxy side chain of a nearby serine residue, and the oxygen in the substrate^16^. The key serine residue is conserved in DmsA, Psr1A, Psr2A, and Psr3A (Fig 3e, **S6a**). The coordinating Ser^205^ is vital for the reduction of DMSO by DmsA (Fig 3f); however, to our surprise, the corresponding serine residues were irrelevant for the sulfate-reducing capacity of Psr1A, Psr2A, and Psr3A (Fig 3g, **S6b**). Even more surprising, the *moaABCDEF* gene products, which catalyze MOCO cofactor synthesis, were dispensable for the activity of the periplasmic sulfate reductase Psr3 (Fig 3g), but not for the reduction of DMSO by DmsABC (Fig 3f). Together, these data demonstrate that, in contrast to canonical DMSO reductase, periplasmic sulfate reductases do not use the MOCO cofactor during catalysis. All A, B, and C subunits were needed for the enzymatic activity of Psr3 (**Fig S6c, S6d**), suggesting that periplasmic sulfate reductases must utilize a redox-active center at the A subunit other than the MOCO prosthetic group. The FS0 is the only other redox center present in the A subunit of the periplasmic sulfate reductases. To visualize whether the FS0 is responsible for the reduction of sulfate, we pursued an electron paramagnetic resonance (EPR) biophysical approach. Membranes isolated from *psr3*^+^ or *psr3* S199G^+^ *Salmonella* strains, bearing deletions in the remaining *dmsA* paralogs, harbored the FS0 EPR signal (Fig 3h, left panel) characteristic of the canonical DmsA protein (**Fig S6e**). The addition of sulfate oxidized the FS0 redox center of the wild-type Psr3 and Psr3 S199G variant, but not the DmsA protein (Fig 3h, right panel). The FS0 of DmsA did, nonetheless, get oxidized by its cognate DMSO electron acceptor (**Fig S6e**). These biophysical findings support the idea that the FS0 of the Psr3 paralog is the site where sulfate reduction takes place in this periplasmic sulfate reductase. To test this model further, we deleted the asparagine residue at position 66, shortening the distance between Cys^A^ and Cys^B^ residues coordinating the FS0, and independently introduced an alanine substitution in the bridge Arg^105^ contributing critical bonding to Cys in the D position (ref^17,18^, Fig 3i, **Fig S6f**). Membranes isolated from a *Salmonella* strain expressing either Psr3 ΔAsn^66^ or Psr3 R105A variants were not only deficient in sulfate reductase activity (Fig 3j, **S6f**) but also lacked the characteristic FS0 EPR signal (**Fig S6g**, bottom panel). Together, these biochemical and biophysical approaches demonstrate that the reduction of sulfate takes place in the FS0 of Psr3. It is likely that these periplasmic sulfate reductases still load MOCO into the ABC complex. Hence, in addition to supporting Psr activity via the FS0, they may also use MOCO for an undiscovered activity.

## Periplasmic sulfate reductases foster sulfur assimilation.

**Figure 4.**
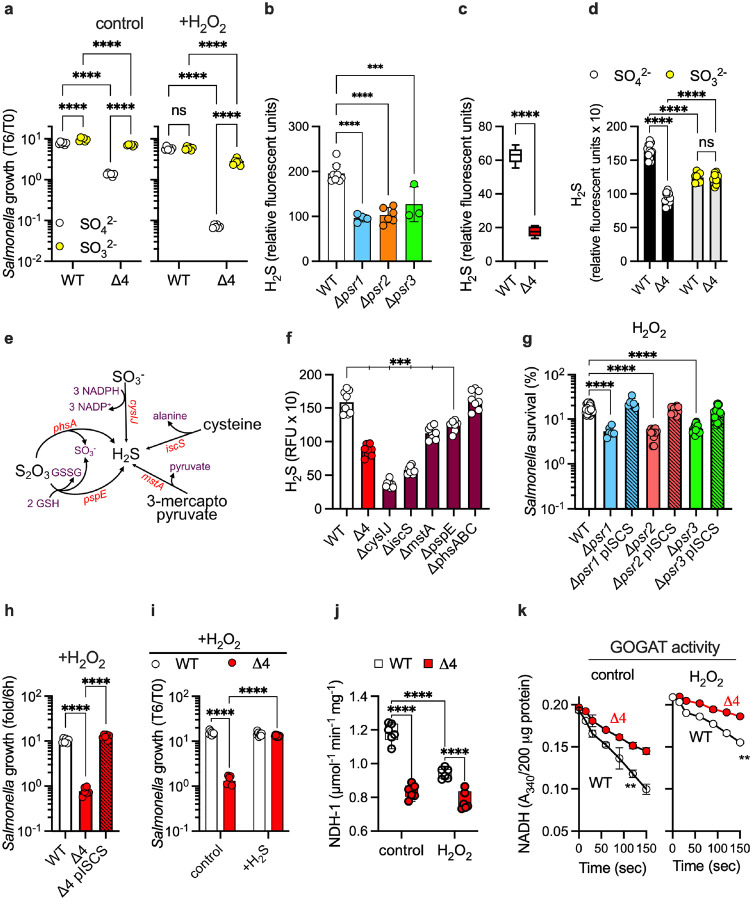
Periplasmic sulfate reductases promote redox balance and resistance to oxidative stress in *Salmonella*. **a.** Susceptibility of *Salmonella* cultured in MOPS-GLC minimal medium supplemented with either sulfate or sulfite was evaluated following treatment with 300 μM H_2_O_2_. Δ4 is a quadruple mutant of all four *dmsA* paralogs. Bacterial survival was assessed by CFU enumeration. The data are the mean ± SD; n = 6 from 2 individual tests. **b,c,d,f.** Quantification of intracellular H_2_S concentrations in *Salmonella* grown in MOPS-GLC minimal medium under ambient air with shaking (**b, c, f**) or standing (**d**). Culture media contained either sulfate (**b,c,d,f**) or sulfite (**d**) as sole sulfur sources. H_2_S was detected using the WSP5 fluorescent (**b, c**) or 7-azido-4-methylcoumarin (**d, f**) probes. Relative fluorescent units were normalized to OD_600_. Data are the mean ± SD from 2–4 independent experiments. (**b,** n=8,6,4,3; **c,** n=6; **d,** SO_4_^2−^ n=12, SO_3_^3−^ n=8; **f,** n=8). **e.** Sulfide production pathway in *Salmonella*. Survival of single (**g**) or quadruple (Δ*4*) (**h**) mutants to after treatment with 200 or 400 μM H_2_O_2_ for 2 and 6 h in PBS (**g**) and EG medium (**h**), respectively. Where indicated, the strains carried the pWSK29 plasmid expressing the *iscS* gene that encodes a cysteine desulfurase. The data are the mean ± SD from 3–10 independent experiments (**g,** n=42,18,18,6,6,12,12; **h,** n=6). **i.** Effect of the addition of 100 μM sulfide (Na_2_S) on the growth of wild-type and Δ*4 Salmonella* in MOPS-GLC-sulfate minimal medium at 37°C without shaking. Some cultures were treated with 100 μM H_2_O_2_. The data represent the mean ± SD from 2–3 individual experiments (control n=8; +H_2_S n=10). **j,** NDH-I dehydrogenase activity in inverted membranes isolated from *Salmonella* grown in MOPS-GLC-sulfate medium was assessed spectrophotometrically by monitoring the oxidation of deamino-NADH. The data are the mean ± SD; n = 6 from 2 independent experiments. **k.** NADH-dependent GOGAT activity in *Salmonella* grown in MOPS-GLC-sulfate medium was measured spectrophotometrically by monitoring NADH oxidation at 340 nm. The data are presented as the mean ± SD; n = 3 from 2 independent experiments. Some specimens were treated with 400 μM H_2_O_2_ for 30 min prior to assay (**j, k**). **, *p* < 0.01; ***, *p* < 0.001; ****, *p* <.0001 by two-way ANOVA (**a, d, g, i, j**), one-way ANOVA (**b, f, h**), or unpaired *t* test (**c, k**).

We next examined how periplasmic sulfate reduction promotes *Salmonella* growth and antioxidant defense. Wild-type and Δ4 strains maintained similar cytoplasmic pH and ATP levels under resting and H_2_O_2_ stress (**Fig S7a**, **S7b**), indicating that these enzymes do not generate ATP via dissimilatory sulfate reduction. The activity of the Psr enzymatic complexes nonetheless helped *Salmonella* maintain redox balance (**Fig S7c**). In the assimilatory pathway, sulfate is reduced to hydrogen sulfide (H_2_S) via a sulfite intermediate. We noticed that *Salmonella* utilizes periplasmic sulfate reductases to boost the intracellular concentration of sulfite (Fig 2i). The addition of sulfite completely rescued the growth defect of untreated or H_2_O_2_-treated Δ*4 Salmonella* (Fig 4a), suggesting that periplasmic sulfate reductases enable sulfite synthesis. Strains of *Salmonella* lacking individual *psr1*, *psr2*, or *psr3* gene clusters synthesized reduced levels of H_2_S compared to wild-type controls (Fig 4b), a defect that was accentuated in the Δ*4* strain lacking all periplasmic sulfate reductases (Fig 4c). Expression of the *psr1*, *psr2*, or *psr3* gene clusters from the low-copy pWSK29 plasmid enhanced the H_2_S-producing capacity of wild-type *Salmonella* (**Fig S8a**). The defective production of H_2_S by the Δ*4 Salmonella* strain is a consequence of its lack of sulfite production, as suggested by the restoration of intracellular H_2_S synthesis upon the addition of sulfite (Fig 4d). The *Salmonella* genome encodes several H_2_S-generating pathways (Fig 4e), one of which, *mstA*, is activated under anaerobiosis (**Fig S8b**). The production of H_2_S by anaerobic *Salmonella* is mediated, in decreasing order of importance by *cysIJ*, *iscS*, *mstA*, and *pspE* gene products (Fig 4f). It is likely that the H_2_S synthesized in the Psr-dependent assimilation of sulfate requires the reduction of sulfite by CysIJ. In support of the idea that Psr proteins help *Salmonella* by enabling H_2_S production, the expression of *iscS*-encoded cysteine desulfurase (a major producer of H_2_S) *in trans* or supplementation of the culture media with an H_2_S donor restored the growth and peroxide resistance of the Δ*4 Salmonella* strain (Fig 4g, 4h, 4i, **S8a**).

Because H_2_S participates in the biosynthesis of [4Fe-4S] prosthetic groups^19^, we compared wild-type and Δ*4 Salmonella* for the activity of NADH dehydrogenase that harbors 8 [Fe-S] clusters^20^. The Δ*4 Salmonella* strain contained lower *nuo*-encoded NADH dehydrogenase activity than wild-type controls (Fig 4j). The Δ*4 Salmonella* strain also harbored lower levels of the [4Fe-4S]-containing glutamate synthase (Fig 4k, **S8c**). By fostering glutamate synthase and NDH-I enzymatic activities, the assimilation of sulfur by the enzymatic activity of periplasmic sulfate reductases likely contributes to the growth and oxidative stress resistance of *Salmonella*^21–23^.

## Discussion

The synthesis of O_2_ by photosynthetic cyanobacteria 2.5 billion years ago brought about the GOE, a period in Earth’s history of profound geochemical changes and a formidable force of evolution, as illustrated by the archaeon and bacterial symbiogenesis that launched eukaryota^24^. In addition to fomenting ATP synthesis in aerobic respiration, O_2_ enabled the synthesis of diverse terminal electron acceptors. Microorganisms exploited the versatility of the molybdenum-dependent DMSO reductase superfamily to exploit the newly formed terminal electron acceptors^9^. Genes of the DMSO reductase superfamily account for 2.2 percent of the *Salmonella* genome and power cellular energetics via the reduction of formate, nitrate, DMSO, methionine sulfoxide, tetrathionate, thiosulfate, and trimethylamine N-oxide. We have discovered that three of the *dmsA* paralogs encode previously unknown assimilatory periplasmic sulfate reductases that support gastrointestinal colonization while also fostering oxidative stress resistance in nontyphoidal *Salmonella*.

The electrochemistry catalyzed by DMSO reductase family members spans more than one volt, allowing organisms to leverage a wide variety of redox substrates^25^. The rich chemistry catalyzed by these reductases is founded on two-electron reactions in which molybdenum cycles between the IV and VI redox states. The catalytic versatility of molybdenum is dictated by the redox environment provided by coordinating pyranopterin dithiolenes and the side chains of neighboring amino acids^14^. Periplasmic sulfate reductases retain the structural fold and bear the conserved coordinating serine residue of canonical DmsA. Despite these conserved features, the catalytic cycle of *Salmonella* periplasmic sulfate reductases does not involve the MOCO cofactor, as demonstrated by the excellent sulfate reductase activity of Prs3 S199G^+^ and MOCO-deficient Δ*moa Salmonella* strains. Instead, sulfate reduction by Psr3 involves the FS0 metal center, as shown by the sulfate-induced changes in EPR spectra. Moreover, Asn^66^ and bridge Arg^105^, located near Cys^A^ and Cys^B^ residues coordinating the FS0 [4Fe-4S] cluster, are vital for sulfate reductase activity. The MOCO-independent function of Psr3A contrast with the FS0-independent functions of the DorA and TorA branch of the MopB superfamily^9^. Orthologs of the *psr* genes are distributed among phylogenetically diverse organisms. Hence, the FS0-based catalytic cycle may be common among MopB family members. Molybdenum is a trace element that may not be readily available in all environments. The ability to utilize alternative cofactors like [4Fe-4S] could allow organisms to thrive in ecological niches where molybdenum is scarce, thus enabling the distribution and evolution of microbes in various habitats.

## METHODS

### Bacterial strains, plasmids, and growth conditions.

*Salmonella enterica* serovar Typhimurium strain 14028s and its derivatives, along with *Escherichia coli strains* and the plasmids used in this study, are listed in **Tables S2** and **S3**. Deletion mutants were constructed using the λ-Red homologous recombination system as previously described^26^. Site-directed mutagenesis was performed using Pfu Ultra High-Fidelity DNA Polymerase (Agilent, Santa Clara, CA) following the manufacturer’s instructions. The primes used in this study are described in **Table S4**. Bacteria were routinely grown in LB broth at 37°C with shaking in ambient air. Where indicated, *Salmonella* was grown in MOPS (morpholinopropanesulfonic acid) minimal medium (40 mM MOPS, 1 mM tricine, 2 mM K_2_HPO_4_, 10 μM FeSO_4_, 9.5 mM NH_4_Cl, 276 μM K_2_SO_4_, 0.5 μM CaCl_2_, 50 mM NaCl, 525 μM MgCl_2_), pH 7.2, supplemented with 0.4% casamino acid (MOPS-CAA) or 0.4% D-glucose (MOPS-glucose [GLC])^27^. Where specified, alternative sulfur sources (Na_2_SO_3_, Na_2_S_2_O_3_, or Na_2_S) were used instead of K_2_SO_4_ to prepare MOPS-sulfite, MOPS-thiosulfate, or MOPS-sulfide media, respectively. Bacteria were grown under aerobiosis in a shaking incubator, anaerobiosis in a Bactron anaerobic chamber (Shel Lab, Cornelius, OR), or hypoxia in tightly closed tubes filled with medium and incubated standing. When appropriate, penicillin, chloramphenicol, tetracycline, and kanamycin were added at the final concentrations of 250, 40, 20, or 50 μg/ml, respectively.

### Animal studies.

All mice were bred according to protocols approved by the Institutional Animal Care and Use Committee (IACUC) at the University of Colorado School of Medicine. Six to 8-week-old C57BL/6 mice were inoculated i.p. with ~300 CFU of *Salmonella* mixtures containing equal numbers of wild-type and mutant strains. The bacterial burden was quantified in livers and spleens 3 days post-infection by plating onto LB agar containing the appropriate antibiotics. Competitive index was calculated as (strain 1/strain 2) output / (strain 1/strain 2) input. For oral infection, a streptomycin-pretreatment colitis model was used as previously described^28^. Briefly, 6 to 8-week-old C57BL/6 mice were deprived of food and water for 4 h prior to treatment with 20 mg of streptomycin by oral gavage. After 4 h, food and water were returned. After 24 h of streptomycin treatment, mice were inoculated orally with 100 μL *Salmonella* mixture containing 3.5x10^8^ CFU of equal numbers of wild-type and mutant strains using gavage needles. The bacterial burden was assessed by enumerating CFUs from the cecum, ileum, colon, mesenteric lymph node (MLN), liver, and spleen 4 days post-infection.

### Macrophage killing assays.

Peritoneal macrophages were isolated from 6- to 8-week-old C57BL/6 and *Cybb*^−/−^ mice 4 days after intraperitoneal injection with 1 mL of filter-sterilized sodium periodate (1 mg/mL in PBS; Millipore Sigma). Macrophages were harvested in RPMI^+^ medium (RPMI medium supplemented with 10% heat-inactivated fetal bovine serum, 1 mM sodium pyruvate, 2 mM L-glutamine, and 20 mM HEPES). Peritoneal exudate cells were collected by centrifugation at 500xg for 10 min and resuspended in RPMI^+^ medium at 3 x10^6^ cells/ mL. One-hundred μL of peritoneal exudate cells were seeded per well in 96-well plates and incubated at 37°C with 5% CO_2_ tissue culture chamber. Confluent peritoneal macrophages were infected at an MOI of 2 with *Salmonella* grown overnight in LB broth at 37°C in a hypoxia chamber (Coy Laboratory Products, Grass Lake, MI) containing 1% O_2_. After 1 h infection, cells were incubated for an additional hour with fresh RPMI^+^ medium containing 50 μg/mL gentamicin to eliminate extracellular bacteria. Medium was then replaced with RPMI^+^ medium containing 10 μg/mL gentamicin. Specimens were lysed in PBS with 0.1% Triton X-100 at T=0, T=2, and T=20, respectively. Lysates were serially diluted in PBS and plated on LB agar. Colony-forming units (CFUs) were enumerated after overnight incubation at 37°C. Fold replication of intracellular *Salmonella* was calculated as CFU 2 h and 20 h postinfection relative to the burden recovered at T=0.

### H_2_O_2_ killing assays.

*Salmonella* grown overnight in LB broth at 37°C in a shaker incubator was diluted to 2x10^6^ CFU/200 μL in PBS and seeded in 96-well plates. The specimens were challenged for 2 h at 37°C with 400 μM of H_2_O_2_. Percent survival was calculated as CFU of the H_2_O_2_-treated / CFU of untreated samples. Where indicated, *Salmonella* grown aerobically overnight in LB broth at 37°C were diluted to approximately 5x10^7^ cells/mL in MOPS-GLC minimum medium. Bacterial cultures were exposed to 100–300 μM H_2_O_2_ under either shaking or hypoxic conditions. At the designated time points, samples were serially diluted and plated on LB agar for CFU enumeration. Bacterial survival was assessed by calculating the fold change in CFU relative to the T=0.

### Anaerobic growth assay.

*Salmonella* grown aerobically overnight in LB broth at 37°C were diluted to ~5x10^7^ cells/mL in either MOPS-CAA or LB medium. Cells were transferred to a Bactron anaerobic chamber (Shel Lab, Cornelius, OR) and treated with 40 mM of the terminal electron acceptors DMSO or L-methionine sulfoxide (both from Millipore Sigma, St. Louis, MO). Cell growth was monitored by measuring OD_600_, or by CFU enumeration. Fold growth was calculated as the ratio of CFUs at T=24 vs T=0.

### Membrane preparation.

The membranes for DMSO or sulfate reductase activity assays and EPR spectroscopy were isolated as previously described^4^. Briefly, *Salmonella* grown overnight anaerobically in LB broth in an anaerobic chamber (Shel Lab, Cornelius, OR) were harvested by centrifugation at 8,000xg for 5 min and resuspended in 3 mL of 50 mM Tris-HCl buffer, pH 8.0, containing 0.1 mg/mL RNase (Millipore Sigma), 0.1 mg/mL DNase (Promega), and 2 mM PMSF (phenylmethylsulfonyl fluoride). Bacterial cultures were disrupted on ice using a probe sonicator (Sonic Dismembrator model 100, Thermo Fisher Scientific) at 40% amplitude with alternating 5- and 10-second pulses for a total sonication time of 30 s. Crude cellular debris was removed by centrifugation at 10,000 x g for 15 min at 4°C. The resulting supernatant was ultracentrifuged at 150,000xg for 1.5 h at 4°C in an optima XL-100K ultracentrifuge (Beckman Instruments, Inc., Fullerton, CA). The membrane-containing pellets were suspended in 20 mM sodium phosphate buffer, pH 6.8. Freshly isolated membranes were used immediately for enzymatic assays.

To measure NADH dehydrogenase I (NDH-I) activity, inverted membranes were isolated as described previously^23^. Briefly, the vesicles were obtained from cells grown aerobically in MOPS-GLC minimal medium to an OD_600_ of ~0.4. Bacteria were pelleted by centrifugation at 10,000xg for 15 min at 4°C, washed twice with ice-cold 50 mM MES buffer, pH 6.0, containing 10% glycerol. The resulting bacterial suspensions were disrupted by sonication in the same buffer. Cell debris was removed by centrifugation at 10,000×g for 15 min at 4°C. Supernatants were spun at 100,000×g for 2 h at 4°C in an optima XL-100K ultracentrifuge (Beckman Instruments). The resulting inverted membrane vesicles were resuspended in ice-cold MES buffer. Protein concentrations were determined using the BCA Protein Assay Kit (Thermo Scientific) according to the manufacturer's instructions.

### Enzymatic assays.

DMSO and sulfate reductase activities were assayed under anaerobic conditions by monitoring the oxidation of benzyl viologen as previously described^4^. Reactions were carried out in a total volume of 1 mL in 20 mM sodium phosphate buffer, pH 6.8, containing 0.5 mM DTT, 0.2 mM benzyl viologen, 100 μg of the reducing agent sodium dithionate, 100 μg freshly prepared membrane protein, and 2 mM DMSO or sulfate. Benzyl viologen was reduced with 150 μM sodium dithionite. The oxidation of benzyl viologen was monitored at 575 nm using a CARY 60 UV-Vis spectrophotometer (Agilent Technologies, Santa Clara, CA) immediately after the addition of DMSO or sulfate. The enzyme activity was calculated using extinct coefficient of benzyl viologen (ε_benzyl viologen_ = 8.9 mM^−1^ cm^−1^).

The activity of the NADH-dependent glutamate synthase (GOGAT) was measured kinetically by monitoring the oxidation of NADH at 340 nm^29^. *Salmonella* cultures grown aerobically overnight in LB broth at 37°C in a shaker incubator were diluted to ~5x10^7^ cells/mL in MOPS-GLC minimal medium supplemented with 250 μM sulfate and cultured at 37°C to OD_600_ of ~0.4. Where indicated, cells were challenged with 400 μM H_2_O_2_ for 30 min. Following treatment, cells were harvested and resuspended in 25 mM HEPES buffer, pH 7.5. The bacterial cells were disrupted by sonication. Cell-free extracts were obtained by centrifugation at 17,000x g for 15 min at 4°C. Protein concentrations were determined using the BCA Protein Assay kit (Thermo Scientific). Enzyme reactions were performed in 25 mM HEPES buffer, pH 7.5, containing 5 mM α-ketoglutarate, 5 mM L-glutamine, 3 mM NADH, and 50 μg of protein. NADH oxidation was monitored over 3 min at 340 nm using a microplate reader (Molecular Device, San Jose, CA).

NADH dehydrogenase I (NDH-I) activity was assessed by monitoring the oxidation of NADH at 340 nm^23^. Reactions were performed in 50 mM MES buffer, pH 6.0, containing 10% glycerol and 200 μM of deamino-NADH (nicotinamide hypoxanthine dinucleotide, Millipore Sigma). NDH-I is known to utilize diamino-NADH and NADH with equal efficiency. To evaluate the direct effect of H_2_O_2_ on NDH-I activity *in vitro*, membrane vesicles isolated from *Salmonella* were exposed to 100 μM H_2_O_2_ for 10 min at 37°C. Enzymatic activity was subsequently measured by tracking the oxidation of deamino-NADH in a microplate reader (Molecular Devices).

### Sulfate consumption.

Membranes and cytosolic fractions were isolated from *Salmonella* grown overnight aerobically in LB broth as described^4^. After ultracentrifugation, sulfate reductase activity was measured in both the membrane and cytosol fractions as described in Enzymatic Assays section. To assess the dependence on quinone electron carriers, 1 mg/mL ubiquinone-10 (coenzyme Q10, Millipore Sigma) or menaquinone-4 (MK-4, Millipore Sigma) were added in place of benzyl viologen. Where indicated, the reactions contained 0.2 mM ATP. After 20 min, sulfate concentration in the reaction mixtures was quantified using a Sulfate test kit (Millipore Sigma) according to the manufacturer’s instructions.

### Measurement of intracellular sulfite.

*Salmonella* cultures grown aerobically overnight in LB broth at 37°C were diluted to ~5x10^7^ cells/mL in 3 mL MOPS-GLC minimum medium supplemented with either 250 μM sulfate or sulfite. Cultures were incubated overnight at 37°C under hypoxic conditions. Cells were harvested by centrifugation at 17,000x*g* for 1 min, and pellets were resuspended in 250 μl of deionized water. Cells lysed by sonication were centrifuged at 17,000xg for 15 min at 4°C. The concentration of sulfite in the resulting supernatants was quantified using sulfite test kit (Millipore Sigma) according to manufacturer’s instructions. Intracellular sulfite concentration was normalized to OD_600_.

### Hydrogen sulfide measurements.

Intracellular H_2_S concentrations in *Salmonella enterica* serovar Typhimurium were assessed using the specific fluorescent probes WSP-5 (Cayman Chemical) or 7-azido-4-methylcoumarin (Millipore Sigma) as previously described^4^. Bacteria were cultured in MOPS-GLC minimum medium containing either 250 μM sulfate or sulfite as the sole sulfur source under aerobic conditions to OD_600_ ~ 0.4 Where indicated, some of the bacteria were grown for 6 h standing. After incubation for 30 min with 10 μM of the designated H_2_S probe, the bacteria were pelleted by centrifugation at 15,000×g for 3 min. After washing once with PBS to remove excess probe, the bacterial pellets were resuspended in 2 mL PBS and incubated at room temperature for 30 min. Fluorescence intensity was measured using a Citole S fluorimeter (Beckman Coulter) at excitation/emission wavelengths of 500/533 nm. Relative fluorescent units were normalized to OD_600_ after subtraction of background fluorescence. Where indicated, H_2_S production was assessed *in vitro* using WSP5 fluorescent probes on membranes isolated from *Salmonella* strains as described above. H_2_S production was normalized per μg of membrane protein.

### RNA isolation and quantitative real-time PCR.

*Salmonella* cultures grown aerobically overnight in LB broth at 37°C were diluted to ~5x10^7^ cells/mL in MOPS-GLC minimal medium supplemented with 250 μM sulfate. Total RNA was extracted from bacterial cultures grown to OD_600_ of ~0.4 under aerobic or anaerobic conditions using the High Pure RNA Isolation Kit (Roche) according to protocols provided by the manufacturer. Complementary DNA (cDNA) was synthesized from 1 μg of purified RNA using Luna Universal Probe qPCR Master Mix (NEB, Ipswich, MA) and 10 μM of N6 random primers (Thermo Fisher Scientific). Quantitative real-time PCR was carried out using 10 μM gene-specific primers, and 10 μM probes containing 5′ 6-carboxyfluorescein and 3′ black-hole quencher 1 modifications. (**Table S4**). Standard curves were generated using PCR-amplified DNA fragments containing target gene sequences. The abundance of transcripts was normalized to internal *rpoD* housekeeping gene.

### EPR spectroscopy.

Electron paramagnetic resonance (EPR) was conducted in 20 mM sodium phosphate buffer, pH 6.8, containing 2 mM sodium dithionite and 10 mg/mL membrane protein preparations isolated as described above. Reactions were conducted in the presence or absence of 50 mM DMSO or sulfate in a Bactron anaerobic chamber (Shel Lab) for 20 min. Samples were flashed-frozen in liquid nitrogen after loading into PTFE tubing. Measurements were performed using a finger dewar and liquid nitrogen. Spectra were recorded using an EMXnano Bruker spectrometer (Billerica, MA) under the following conditions: 77 K temperature, 10 mW microwave power, 9.65 GHz microwave frequency, and 6 G modulation amplitude. Traces are the average of 10 scans.

### ΔpH measurements.

Cytoplasmic pH was assessed using the ratiometric, pH-sensitive GFP pHluorin derivative that was expressed from the pBAD promoter as previously described^4,30^. Briefly, *Salmonella* harboring the pHIuorin plasmid was grown aerobically overnight in LB broth supplemented with 0.2% arabinose and 100 μg/mL penicillin. Cultures were diluted to 2x10^8^ cells/ml in MOPS-GLC minimum medium containing either 250 μM sulfate or sulfite, or 100 μM sulfide and equilibrated for 10 min at 37°C. Fluorescence spectra (excitation at 300–490 nm, emission at 510 nm) were recorded for 4 min using a Shimadzu R5300C spectrofluorometer (Shimadzu Corporation, Kyoto, Japan). Bacterial cells were challenged with 400 μM H_2_O_2_, and spectra were monitored for 25 min. To determine the cytoplasmic pH values, fluorescence ratios (405/488 nm) were calibrated using cells equilibrated in MOPS GLC media, pH 5.0 to 8.5, in the presence of protonophore potassium benzoate. Internal pH was calculated using a Boltzmann sigmoid best-fit curve.

### ATP quantification.

Intracellular ATP levels were measured using a luciferase-based ATP Determination Kit (Molecular Probes, Thermo Fisher Scientific). *Salmonella* grown aerobically overnight in LB broth at 37°C were diluted to ~5x10^7^ cells/ml in MOPS-GLC minimum medium supplemented with either 250 μM sulfate or sulfite. Bacterial cultures were incubated in a 1% O_2_ hypoxic chamber for 4 h at 37 °C. Where specified, some of the specimens were treated for 30 min with 400 μM H_2_O_2_. Bacteria were harvested by centrifugation at 15,000xg for 3 min. ATP in bacteria was extracted in 0.5 mL of ice-cold 380 mM formic acid containing 17 mM EDTA. The resulting specimens were pelleted for 1 min at 16,000xg. Supernatants were diluted 25-fold into a 100 mM N-tris(hydroxymethyl)methyl-2-aminoethanesulfonic acid (TES) buffer, pH 7.4. Ten microliters of samples or ATP standards were combined with 90 μl of a master mix consisting of 8.9 mL of water, 500 μL of 20X buffer (200 mM Tris, pH 7.5, 2 M NaCl, 20 mM EDTA, and 0.2% Triton X-100), 500 μL of 10 mM D-luciferin, 100 μL of 100 mM dithiothreitol (DTT), and 2.5 μL of 5 mg/ml of firefly luciferase. The specimens were incubated for 5 min at room temperature protected from light. Luminescence was measured using an Infinite 200 PRO instrument (Tecan Life Sciences). ATP concentrations, determined by linear regression using ATP standards, were normalized to CFU/mL.

### NADH and NAD^+^ measurements.

Intracellular concentrations of NADH/NAD^+^ were determined as previously described^4^. *Salmonella* cultured aerobically overnight in LB broth at 37°C were diluted to ~5x10^7^ cells/mL in MOPS-GLC minimal medium. Cells were cultured aerobically in MOPS-GLC minimal medium at 37°C to OD_600_ of ~0.4. NADH and NAD^+^ were separately extracted from cell pellets obtained from 1.6 mL cultures using 120 μL of 0.2 M NaOH and 0.2 M HCl, respectively. Extracts were neutralized by the addition of equal volumes of 0.1 M HCl (for NADH) or 0.1 M NaOH (for NAD^+^). Ten microliters of the neutralized extracts were added to a 90 μL reaction containing 100 mM bicine (pH 8.0), 4 mM EDTA, 1.6 mM phenazine methosulfate, 0.42 mM 3-(4,5-dimethylthiazol-2-yl)-2,5-diphenyltetrazolium bromide, and 10% ethanol. Reactions were initiated by adding 100 μL of 0.4 μg alcohol dehydrogenase (Millipore Sigma). Absorbance was measured spectrophotometrically at 570 nm using a microplate reader (Molecular Device, San Jose, CA). NADH and NAD^+^ concentrations were calculated based on standard curves and normalized to OD_600_.

### Bioinformatics.

To identify the evolutionary relationships among DMSO reductase family members encoded by the *Salmonella enterica* serovar Typhimurium 14028s genome, a sequence-based phylogenetic analysis was performed. The amino acid sequence of DMSO reductase subunit A (DmsA) from *S. Typhimurium* 14028s was used as the query in a BLASTp search against the *Salmonella* (TaxID: 588858) non-redundant protein database. To enhance detection of remote homologues, the same amino acid sequences were queried in DELTA-BLAST (Domain Enhanced Lookup Time Accelerated BLAST). Both searches were conducted using default parameters on the NCBI BLAST site (https://blast.ncbi.nlm.nih.gov/). The combined output was manually curated to remove redundant entries, yielding a set of 17 unique DMSO reductase-like proteins. Amino acid sequences of the identified proteins were retrieved in FASTA format from the NCBI protein database. Multiple sequence alignment was performed using MUSCLE (v3.8) with default settings. Phylogenetic analysis was carried out using the Neighbor-Joining method, as implemented in MEGAx(v10.2.6)^31^. Evolutionary distances were computed using the Poisson correction method, which estimates the number of amino acid substitutions per site. The reliability of the inferred tree was assessed by a bootstrap test with 500 replicates. All ambiguous positions were eliminated using pairwise deletion. The resulting phylogenetic tree was visualized in circular format and drawn to scale, with branch lengths proportional to evolutionary distance.

The cross-species phylogenetic analysis of DMSO-reductase catalytic subunits was built from amino-acid sequence queries retrieved from NCBI using the five DmsA paralogs from *Salmonella enterica* serovar Typhimurium 14028s. The queries were subjected to JackHMMER (HMMER v3.4) against a comprehensive prokaryotic protein database. Searches were run for five iterations with stringent inclusion thresholds (sequence E-value ≤10^−4^; hit E-value ≤10^−2^). Resulting hits were curated to retain full-length proteins within the expected size range, verified for Mo-bis-PGD domain architecture, filtered and obvious CISM paralogs (NarG, TorA/TtrA, PsrA, FdhG) were excluded unless explicitly used as outgroups. To reduce redundancy, sequences were clustered using CD-HIT at 90% identity (longest representative retained) and selected for *dmsABC* operon context (orthology). Multiple sequence alignment of the resulting >1800 sequences was performed with MAFFT v7.515 (L-INS-i, --localpair --maxiterate 1000), followed by objective trimming with trimAl v1.4 (-gappyout). The alignment was visually inspected in Jalview v2.11.2 to remove residual poorly aligned segments. For paralogous rooting, four *Desulfurococcus* MopB paralogs (TorA/TtrA-like) were included as outgroups; the ML tree was rooted on the outgroup clade, and outgroup sequences were subsequently pruned to obtain a rooted DmsA ingroup topology. Trees were visualized in MEGA X v11 and exported for figure preparation. For sequence selection and divergence-time estimation, a taxonomically representative subset of DmsA orthologs (retaining all five Salmonella DmsA paralogs) was utilized. The timetree was inferred using RelTime-ML implemented in MEGA X, with branch lengths estimated under the substitution model (LG+Γ+F) and gamma-distributed among-site rate heterogeneity. Divergence times were calibrated using soft, wide Min/Max bounds on clean, two-taxon sister splits present in the gene tree and supported by operon context: (i) *Escherichia coli*–*Salmonella enterica* (same DmsA ortholog), (ii) *Salmonella* “3103”–*Enterobacter* “3103” (matched paralog), (iii) *Clostridium*–*Propionispira* as a deeper secondary anchor with wide bounds, and (iv) a *Proteus*–*Providencia* sister pair within Enterobacterales^32^. Calibration densities and time boundaries were implemented using 95% confidence intervals for each internal node^33^. The estimated log-likelihood of the final tree was –21,865.39.

Geochemical trends of environmental sulfur (S), molybdenum (Mo), and oxygen (O_2_) concentrations across Earth's history were extracted from Anbar et al.^34^. The data were digitized and smoothed using the *potrace* library in R. These geochemical traces were overlaid over the molecular timetree to contextualize gene divergence in relation to redox evolution and trace metal bioavailability.

### **Structural modeling**.

The five DmsA paralogs in *S. Typhimurium* 14028s were selected for structural analysis. Predicted 3D structures of all five proteins were generated using AlphaFold3 via the AlphaFold Protein Structure Database or locally implemented in ColabFold with default settings. The top-ranked predicted structure for each protein, based on pLDDT scores, was used for downstream analysis. Electrostatic surface potentials were computed in UCSF ChimeraX (v1.6)^35^ using the Coulombic Surface Coloring tool. Default dielectric parameters were applied. The surfaces are colored from red (negative potential) to blue (positive potential), highlighting charge distributions across the solvent-accessible surfaces. All five protein structures were oriented consistently to facilitate visual comparison. Pairwise sequence similarity between DmsA and each of the other four proteins was calculated using the EMBOSS Needle tool with default gap penalties and the BLOSUM62 matrix. Percentage identity values are annotated in blue text on the figure next to each corresponding structure. For the predicted Mo-bis PGD binding sites and structural model of Psr3 (Fig. 3f, 3j), cofactor binding sites were modeled by structurally aligning the predicted protein models to the crystal structure of *E. coli* DmsA (PDB ID: 1EU1) using the MatchMaker tool in ChimeraX. Coordinates of the molybdenum-bis pyranopterin guanosine dinucleotide (Mo-bis PGD) cofactor and proximal Fe–S clusters were extracted from the reference structure and fitted into the homologous binding clefts in DmsA and Psr3A. Key coordinating residues were identified based on geometric proximity (<4 Å) and canonical motifs conserved in the DMSO reductase family. Interacting residues were visualized in stick representation, and hydrogen bonding or salt bridge interactions were annotated.

### Statistical analysis.

Statistical analysis was performed using GraphPad Prism version 10.4. One-way, two-way ANOVA, and *t*-test were performed with p < 0.05 as the cutoff for statistical significance. Error bars indicate mean ± SD.

## Supplementary Files

This is a list of supplementary files associated with this preprint. Click to download.

- Kimetalsupplementaryinformation.pdf
- S5.xlsx

## Data Availability

Data and materials generated during these investigations are available from the corresponding author upon request.

## References

[1] Winter S. E. Gut inflammation provides a respiratory electron acceptor for Salmonella. Nature 467, 426–429 (2010). 10.1038/nature09415 20864996 PMC2946174

[2] Liou M. J. Host cells subdivide nutrient niches into discrete biogeographical microhabitats for gut microbes. Cell Host Microbe 30, 836–847 e836 (2022). 10.1016/j.chom.2022.04.012 35568027 PMC9187619

[3] Gennaris A. Repairing oxidized proteins in the bacterial envelope using respiratory chain electrons. Nature 528, 409–412 (2015). 10.1038/nature15764 26641313 PMC4700593

[4] Kim J. S. Anaerobic respiration of host-derived methionine sulfoxide protects intracellular Salmonella from the phagocyte NADPH oxidase. Cell Host Microbe 32, 1–14 (2024). 38307020 10.1016/j.chom.2024.01.004 PMC11396582

[5] Unden G. & Bongaerts J. Alternative respiratory pathways of Escherichia coli: energetics and transcriptional regulation in response to electron acceptors. Biochim Biophys Acta 1320, 217–234 (1997). 10.1016/s0005-2728(97)00034-0 9230919

[6] Rivera-Chavez F. Depletion of Butyrate-Producing Clostridia from the Gut Microbiota Drives an Aerobic Luminal Expansion of Salmonella. Cell Host Microbe 19, 443–454 (2016). 10.1016/j.chom.2016.03.004 27078066 PMC4832419

[7] Kim J. S. Oxidative stress activates transcription of Salmonella pathogenicity island-2 genes in macrophages. J Biol Chem 298, 102130 (2022). 10.1016/j.jbc.2022.102130 35714768 PMC9270255

[8] Valvano M. A. Bacterial conversion of a host weapon into a nutritional signal. J Biol Chem 298, 102600 (2022). 10.1016/j.jbc.2022.102600 36244456 PMC9637811

[9] Wells M., Kim M., Akob D. M., Basu P. & Stolz J. F. Impact of the Dimethyl Sulfoxide Reductase Superfamily on the Evolution of Biogeochemical Cycles. Microbiol Spectr 11, e0414522 (2023). 10.1128/spectrum.04145-22 36951557 PMC10100899

[10] Fink R. C. FNR Is a Global Regulator of Virulence and Anaerobic Metabolism in Salmonella enterica Serovar Typhimurium (ATCC 14028s). J Bacteriol 189, 2262–2273 (2007). 17220229 10.1128/JB.00726-06 PMC1899381

[11] Pollock J. D. Mouse model of X-linked chronic granulomatous disease, an inherited defect in phagocyte superoxide production. Nat Genet 9, 202–209 (1995). 7719350 10.1038/ng0295-202

[12] Mastroeni P. Antimicrobial actions of the NADPH phagocyte oxidase and inducible nitric oxide synthase in experimental salmonellosis. II. Effects on microbial proliferation and host survival in vivo. J Exp Med 192, 237–248 (2000). 10899910 10.1084/jem.192.2.237 PMC2193252

[13] Meganathan R. & Kwon O. Biosynthesis of Menaquinone (Vitamin K2) and Ubiquinone (Coenzyme Q). EcoSal Plus 3 (2009). 10.1128/ecosalplus.3.6.3.3 PMC4172378 26443765

[14] Grein F., Ramos A. R., Venceslau S. S. & Pereira I. A. Unifying concepts in anaerobic respiration: insights from dissimilatory sulfur metabolism. Biochim Biophys Acta 1827, 145–160 (2013). 10.1016/j.bbabio.2012.09.001 22982583

[15] Canfield D. E., Habicht K. S. & Thamdrup B. The Archean sulfur cycle and the early history of atmospheric oxygen. Science 288, 658–661 (2000). 10.1126/science.288.5466.658 10784446

[16] Cheng V. W. & Weiner J. H. S- and N-Oxide Reductases. EcoSal Plus 2 (2007). 10.1128/ecosalplus.3.2.8 26443585

[17] Rothery R. A., Stein B., Solomonson M., Kirk M. L. & Weiner J. H. Pyranopterin conformation defines the function of molybdenum and tungsten enzymes. Proc Natl Acad Sci U S A 109, 14773–14778 (2012). 10.1073/pnas.1200671109 22927383 PMC3443133

[18] Burgmayer S. J. N. & Kirk M. L. Advancing Our Understanding of Pyranopterin-Dithiolene Contributions to Moco Enzyme Catalysis. Molecules 28 (2023). 10.3390/molecules28227456 PMC10673323 38005178

[19] Lill R. Function and biogenesis of iron-sulphur proteins. Nature 460, 831–838 (2009). 10.1038/nature08301 19675643

[20] Friedrich T., Dekovic D. K. & Burschel S. Assembly of the Escherichia coli NADH:ubiquinone oxidoreductase (respiratory complex I). Biochim Biophys Acta 1857, 214–223 (2016). 10.1016/j.bbabio.2015.12.004 26682761

[21] Kant S. Gre factors help Salmonella adapt to oxidative stress by improving transcription elongation and fidelity of metabolic genes. PLoS Biol 21, e3002051 (2023). 10.1371/journal.pbio.3002051 37014914 PMC10072461

[22] Margolis A. Arginine Metabolism Powers Salmonella Resistance to Oxidative Stress. Infect Immun 91, e0012023 (2023). 10.1128/iai.00120-23 37191509 PMC10269097

[23] Chakraborty S. Glycolytic reprograming in Salmonella counters NOX2-mediated dissipation of DeltapH. Nat Commun 11, 1783 (2020). 10.1038/s41467-020-15604-2 32286292 PMC7156505

[24] Speijer D. Alternating terminal electron-acceptors at the basis of symbiogenesis: How oxygen ignited eukaryotic evolution. Bioessays **39** (2017). 10.1002/bies.201600174 28054713

[25] Schoepp-Cothenet B. On the universal core of bioenergetics. Biochim Biophys Acta 1827, 79–93 (2013). 10.1016/j.bbabio.2012.09.005 22982447

[26] Datsenko K. A. & Wanner B. L. One-step inactivation of chromosomal genes in Escherichia coli K-12 using PCR products. Proc Natl Acad Sci U S A 97, 6640–6645 (2000). 10829079 10.1073/pnas.120163297 PMC18686

[27] Neidhardt F. C., Bloch P. L. & Smith D. F. Culture medium for enterobacteria. J Bacteriol 119, 736–747 (1974). 10.1128/jb.119.3.736-747.1974 4604283 PMC245675

[28] Jones-Carson J., Husain M., Liu L., Orlicky D. J. & Vazquez-Torres A. Cytochrome bd-Dependent Bioenergetics and Antinitrosative Defenses in Salmonella Pathogenesis. mBio 7 (2016). 10.1128/mBio.02052-16 PMC5181779 27999164

[29] Chen F. L. & Cullimore J. V. Two Isoenzymes of NADH-dependent Glutamate Synthase in Root Nodules of Phaseolus vulgaris L: Purification, Properties and Activity Changes during Nodule Development. Plant Physiol 88, 1411–1417 (1988). 10.1104/pp.88.4.1411 16666475 PMC1055773

[30] Miesenbock G., De Angelis D. A. & Rothman J. E. Visualizing secretion and synaptic transmission with pH-sensitive green fluorescent proteins. Nature 394, 192–195 (1998). 10.1038/28190 9671304

[31] Kumar S., Stecher G., Li M., Knyaz C. & Tamura K. MEGA X: Molecular Evolutionary Genetics Analysis across Computing Platforms. Mol Biol Evol 35, 1547–1549 (2018). 10.1093/molbev/msy096 29722887 PMC5967553

[32] Kumar S. TimeTree 5: An Expanded Resource for Species Divergence Times. Mol Biol Evol 39 (2022). 10.1093/molbev/msac174 PMC9400175 35932227

[33] Tao Q., Tamura K., F U. B. & Kumar S. A Machine Learning Method for Detecting Autocorrelation of Evolutionary Rates in Large Phylogenies. Mol Biol Evol 36, 811–824 (2019). 10.1093/molbev/msz014 30689923 PMC6804408

[34] Anbar A. D. Oceans. Elements and evolution. Science 322, 1481–1483 (2008). 10.1126/science.1163100 19056967

[35] Meng E. C. UCSF ChimeraX: Tools for structure building and analysis. Protein Sci 32, e4792 (2023). 10.1002/pro.4792 37774136 PMC10588335

